# Food words distract the hungry: Evidence of involuntary semantic processing of task-irrelevant but biologically-relevant unexpected auditory words

**DOI:** 10.1371/journal.pone.0190644

**Published:** 2018-01-04

**Authors:** Fabrice B. R. Parmentier, Antonia P. Pacheco-Unguetti, Sara Valero

**Affiliations:** 1 Neuropsychology & Cognition Group, Department of Psychology and Research Institute for Health Sciences (iUNICS), University of the Balearic Islands, Palma, Balearic Islands, Spain; 2 Balearic Islands Health Research Institute (IdISBa), Palma, Balearic Islands, Spain; 3 School of Psychology, University of Western Australia, Perth, Western Australia, Australia; 4 Centro de Psicología Pacheco Unguetti, Granada, Spain; Universidad de Salamanca, SPAIN

## Abstract

Rare changes in a stream of otherwise repeated task-irrelevant sounds break through selective attention and disrupt performance in an unrelated visual task by triggering shifts of attention to and from the deviant sound (deviance distraction). Evidence indicates that the involuntary orientation of attention to unexpected sounds is followed by their semantic processing. However, past demonstrations relied on tasks in which the meaning of the deviant sounds overlapped with features of the primary task. Here we examine whether such processing is observed when no such overlap is present but sounds carry some relevance to the participants’ biological need to eat when hungry. We report the results of an experiment in which hungry and satiated participants partook in a cross-modal oddball task in which they categorized visual digits (odd/even) while ignoring task-irrelevant sounds. On most trials the irrelevant sound was a sinewave tone (standard sound). On the remaining trials, deviant sounds consisted of spoken words related to food (food deviants) or control words (control deviants). Questionnaire data confirmed state (but not trait) differences between the two groups with respect to food craving, as well as a greater desire to eat the food corresponding to the food-related words in the hungry relative to the satiated participants. The results of the oddball task revealed that food deviants produced greater distraction (longer response times) than control deviants in hungry participants while the reverse effect was observed in satiated participants. This effect was observed in the first block of trials but disappeared thereafter, reflecting semantic saturation. Our results suggest that (1) the semantic content of deviant sounds is involuntarily processed even when sharing no feature with the primary task; and that (2) distraction by deviant sounds can be modulated by the participants’ biological needs.

## Introduction

Efficient functioning often requires the ability to focus on a task while filtering out irrelevant stimuli. Yet it is adaptive for our attention filters to remain partially permeable and allow the detection of sudden and potentially important changes in our immediate environment that may call for a modification of our behavior. The trade-off between selective attention and change detection affords behavioral flexibility but may at times come at a cost (distraction) when our attentional filters let through stimuli that are of no relevance. One class of stimuli known to yield this type of distraction is unexpected changes in our auditory surroundings (deviant sounds). Deviant sounds are stimuli that violate the predictions of our cognitive system by deviating from an otherwise repetitive or structured sequence, be it on the basis of sensory features [1–6], rules [7–14], or self-generated predictions [15–17].

Deviant sounds elicit well-defined electrophysiological responses marking the detection of a mismatch between an incoming sound and sensory predictions, the involuntary orientation of attention towards the deviant sound, and the reorientation of attention toward the primary task [18–23]. At the behavioral level, deviant sounds delay (and sometimes reduce the accuracy of) responses to target stimuli [24], whether presented in purely auditory [3,25–27], visual [26,28,29], or in cross-modal oddball tasks where irrelevant and target stimuli are presented in distinct sensory modalities: auditory-visual [28,30–42] or tactile-visual [43,44]. This distraction effect reflects the deviant sound’s violation of predictions rather than its low frequency of occurrence per se [8,10,12,13], and results from the shift of attention to the deviant sound and reorientation to the target stimulus [2,3], and from the temporary suspension and resetting of action plans [45,46].

### The semantic aftermath of deviance distraction

There is good evidence suggesting that deviant sounds undergo some level of automatic semantic processing (e.g., [47–49]). Electrophysiological studies suggest that semantic features can modulate early electrophysiological responses to a novel sound regardless of whether participants attend it or not [48,50]. For example, the MMN response (a negative electrophysiological deflection observed in response to rare and unexpected acoustic changes in an otherwise repeated or predictable sequence of auditory stimuli, typically observed 100 to 150 following the deviant sounds’ onset [4,23,51]) is modulated by deviant sounds with a sexual connotation [48]. Also, the P3a response (marking the involuntary orientation of attention towards deviant or novel sounds [52,53]) has been found to vary with the novel sounds’ identifiability [54] or aversive emotional valence [47]. Finally, the P3a response to a deviant word (e.g., “duck”) was found to decrease when a deviant sound is primed by another deviant stimulus consisting of its associated sound (e.g., “quack”, [55]). The involuntary appraisal of the deviant sounds’ semantic features can also affect behavioral performance in a primary task [49,56,57]. For example, using a task in which participants categorized the direction of visual arrows preceded by task-irrelevant sounds, Parmentier [56] reported that deviant sounds “left” and “right”, while distracting participants by virtue of violating the pattern of standard tones, modulated response times as a function of their relationship with the visual targets (congruent or incongruent).

Parmentier [24] argued that deviant sounds affect behavioral performance through two distinct effects (deviance distraction and the semantic effect) and that although the second is in part contingent upon the first [57], it follows, once triggered, its own independent course. This proposition is backed up by a pattern of double dissociation between the two effects. Indeed, on the one hand deviance distraction, but not the semantic effect, reduces with task practice and increases as the number of dimensions (acoustics, lexicality, source) differentiating the standard from the deviant sound increases [56]. On the other hand, Parmentier, Turner and Elsley [58] reported that the semantic effect, but not deviance distraction, increased when the temporal interval between irrelevant sound and target increased (allowing more time for sound-related semantic activations to build up prior to the onset of the visual target). The semantic effect, just like deviance distraction, is modulated by predictability, however. Indeed, Parmentier and Kefauver [59] manipulated the proportion of congruent deviant sounds “left” or “right” (.2, .5, or .8) in a left/right visual arrow categorization task and showed that participants built corresponding predictions. While the deviant sounds yielded deviance distraction in all conditions, the semantic effect (incongruent vs. congruent trials) was modulated by the proportion of congruent trials: it was eliminated when congruent trials were rare (for response times to incongruent trials were shortened by the predictability of the incongruence and response times to congruent trials, in contrast, were lengthened by the fact that the congruence violated predictions) and maximum when congruent trials were most frequent (for response times to incongruent trials were longer due to a combination of crosstalk interference and the violation of predictions and response times to congruent trials were reduced due as a result of congruency and its predictability).

### Can semantic processing be demonstrated for task-irrelevant but biologically-relevant information?

The studies described in the previous section suggest that deviant sounds undergo some involuntary semantic processing and that the outcome of this processing can in turn impact on performance in the primary task [24,56,57,59]. In these studies, measuring the impact of the sounds’ semantic processing was achieved through the prism of the semantic congruence effect. Sounds were task-irrelevant in the sense that participants were instructed to ignore them and that the sounds had equal probabilities to be congruent or incongruent [except in, 59, where this factor was purposely manipulated]. Yet one cannot rule out the possibility that the semantic processing of the deviant sounds “left” and “right” was influenced by the tuning of the cognitive system to process left/right information in the primary task. Such overlap appears methodologically necessary as there would be no way of establishing whether, for example, the meaning of a deviant word such as “cake” is processed in a left/right categorization task. Or would there?

The aim of our study was to examine whether deviant sounds undergo semantic analysis when they do not overlap with the primary task’s characteristics but, instead, relate to the participants’ biological needs. The psychology literature contains numerous demonstrations that one’s individual characteristics or state can influence how our attention is deployed and how we process information. For example, participants in an induced state of sadness or happiness are more susceptible to distraction by deviant sounds than participants in a neutral emotional state [60,61], and participants in a state of fear present with an enhanced sensory sensitivity [62]. Evidence also shows that anxious individuals are more vigilant to threat-related stimuli than non-anxious individuals [63], a finding also observed in individuals in a heightened state of anxiety [64]. Phobia-related stimuli are especially potent at capturing attention in phobic individuals [65,66], and evidence indicates that anxious individuals experience greater difficulty in disengaging from fear-related stimuli than non-anxious individuals. Additional evidence includes the finding that an increase in one’s state of subjective appetite is accompanied by a bias of attention towards food-related stimuli [67], or that substance-related stimuli are experienced as especially salient and attention-gabbing by substance users [68]. Finally, evidence suggests that food-related thoughts in participants experiencing food cravings consume cognitive resources, as indicated by slower responses in simple reaction time tasks [69,70] and an attentional bias toward food-related cues [71]. Thus, there is ample evidence indicating that attentional deployment is influenced by one’s motivations, psychological characteristics or state, and biological needs.

The idea underpinning our study was straightforward: We aimed to test the extent to which hungry and satiated participants would differ in their processing of task-irrelevant deviant sounds relating to food (relative to control words). Our hypothesis was simple: if the deviant sounds’ semantic processing fulfills the general adaptive function of exploring and assessing changes in our environment for their potential general relevance to us, then food-related deviant words should distract hungry participants from an unrelated primary task more than it should satiated participants. We expected the differential effect of food-related deviant words in hungry and satiated participants to be most pronounced at the beginning of the task for two reasons. First, deviance distraction effects have been documented to be strongest early in the task and to decrease thereafter [56,72]. Second, there is evidence that participants become temporarily desensitized to a word’s meaning as its activation is repeatedly triggered, a phenomenon known as semantic satiation [73–77] already described a century ago by Titchener [78] and others [79]. Two theoretical accounts have been proposed to explain this effect: Some have argued that it reflects fatigue or adaptation in the neural processing underlying meaning *per se* [73,74], while others have argued that such adaptation occurs at the level of the associations between lexical and semantic codes [80]. There is nevertheless some evidence indicating that perceptual features can enhance the effect, as semantic satiation is more pronounced when words are repeated by the same speaker [76]. The implication for our study is clear and takes the form of a specific prediction: the differential distractive effect between food-related and control deviant words (i.e., an eminently semantic difference) in our oddball task should be most visible at the very beginning of the task and dissipates thereafter. For this reason, we regard (1) the comparison of the two types of deviants in the first block of the experiment and (2) the comparison of this difference between the first and last blocks of our task as especially relevant and the most sensitive tests of the hypothesis that hungry and satiated participants should exhibit different patterns of behavioral responses to these two types of deviant words early on in the task.

## Materials and methods

### Participants

Forty-eight healthy undergraduate students from the University of the Balearic Islands, aged 18 to 49 years (M = 21,08, SD = 5,56; 9 males), took part in the study in exchange for a small honorarium (10 EUR). Only participants reporting no history of an eating disorder, glucose metabolism disorder, or currently undertaking a weight-loss diet were included in the sample.

### Food craving measures

The Spanish versions of the Trait and State Food Cravings Questionnaires [FCQ-T and FCQ-S respectively, 81] were used to assess the intensity of the participants’ food craving along 9 trait and 5 state dimensions. The FCQ-T includes 39 items (e.g., “Food cravings invariably make me think of ways to get what I want to eat”, “I feel I have food on my mind all the time”, “I daydream about food”) that measure trait craving on 6-point Likert scales ranging from 1 (‘Never’ or ‘Not applicable’) to 6 (‘Always’). The FCQ-S consists of 15 items measuring one’s current food craving (e.g., “I have an urge for one of more specific foods”, “I am hungry”) on 5-point Likert scales ranging from 1 (‘Strongly agree’) to 5 (‘Strongly disagree’).

### Procedure

Participants who enrolled for this study were contacted prior to the laboratory session and informed that they had been randomly assigned to one of two experimental groups: satiated vs. food-deprived. Experimental sessions were scheduled at 9am (i.e., after breakfast time) or 3pm (after lunch time). Participants in the satiated group were told that their participation required eating breakfast or lunch (depending on the appointment) in the 60 min prior to taking part in the experiment. Participants in the food-deprived group were asked to abstain from eating lunch if they scheduled to come to the laboratory at lunch time, or to skip breakfast if their appointment was scheduled at 9am. They were allowed to consume water. Participants were told that random saliva tests would be carried out at the end of the experiment to control their food-deprivation/satiation state. Cotton swabs and test tubes were on display in the laboratory where the experiment took place but no saliva sample was actually taken from any of the participants. The subterfuge was used to increase the participants’ compliance with the instructions. Participants indicated explicitly their agreement to take part at that stage and to follow the instructions aforementioned.

Upon arrival at the laboratory, participants provided written informed consent before the testing began. Food items (smoothies, chocolate milk and chocolate bars) were on display at the table where participants sat. They were told that they would be allowed to consume or take them away after the experiment as an extra reward for their participation. Participants first filled in pen-and-paper questionnaires. They were first asked a few questions aimed to confirm that they met the exclusion criteria (no history of eating disorder, glucose metabolism disorder, or currently being on a weight-loss diet), and to indicate how much time had elapsed since their last meal. Participants then filled out the FCQ-T and FCQ-S questionnaires before rating their current state of hunger (from 1, “not at all hungry”, to 9, “Very hungry”). Participants then undertook the cross-modal oddball task described in the next section, before completing the FCQ-S a second time. Finally, all participants were presented with 5 pictures corresponding to the food words used as distractors in the oddball task and asked to rate how much they liked the food corresponding to those words, and how much they would like to eat it at the present time (both using Likert scales ranging from 1, “Not at all”, to 9, “Very much”). Participants were then debriefed and allowed to consume or take with them the smoothies, juices and chocolate bars. The whole testing session lasting approximately 50 minutes. The study was approved by the Bioethical Committee of the University of the Balearic Islands.

### Cross-modal oddball task

Participants were presented with 4 blocks of 168 trials each (8 practice trials followed by 160 test trials). In each trial, participants had to categorize a digit (1–8) presented at the center of the screen as odd or even while ignoring an irrelevant sound presented shortly before that digit. Each trial consisted of the followed sequence of events. A white fixation cross appeared at the center of the black screen and was accompanied by the presentation of a task-irrelevant sound for a duration of 400ms. The fixation cross was then replaced by a target digit (presented in white) remaining on the screen for 150ms, after which it was replaced by the fixation cross during an interval of 750ms. Participants therefore had a total response window of 900ms. At the end of this interval, the next trial started automatically. Participants used the keys Z and X on a Spanish computer keyboard to respond using two fingers of their dominant hand. The mapping between the response keys and the odd/even responses was counterbalanced across participants.

Trials from three sound conditions (standard sound condition, food-related deviant condition, control deviant condition) were mixed in a quasi-random order of presentation within each of four blocks of trials. In the standard condition (87.5% of trials), the sound was a 600Hz sinewave tone lasting 400ms (including 10 ms of rise/fall times), hereafter referred to as the standard sound. In the food-related word condition (6.25% of trials), we used 5 food-related words: “bombón” (chocolate), “helado” (ice cream), “natillas” (custard), “pastel” (cake) and “tarta” (tart). In the control word condition (6.25% of trials), we used five house-hold object words: “cajón” (drawer), “perchero” (coat stand), “persiana” (shutter), “cristal” (window pane), and “manta” (blanket). All words were digitally recorded in the same female voice, normalized, and edited to a duration of 400ms (while maintaining pitch). Each of these words was used twice within each block. The two sets of words were selected from a Spanish database designed to identify words suitable for experimental studies in the field of eating disorders [82] and presented similar subjective frequency and familiarity ratings. A posteriori analysis of the two sets of words using EsPal [83] confirmed their similarity with respect to log-frequency, familiarity, number of syllables and concreteness values. The words and their characteristics are presented in Table 1. The sounds were presented binaurally through headphones at an intensity of approximately 70 dB SPL. A different quasi-random order of presentation of the standard, food-related word and control word trials was used for every participant, with the constraint that deviant trials (food-related or control) never occurred on consecutive trials. Target digits (1–8) were used equally often across the task, and in equal proportions for each of the sound stimuli.

**Table 1:** Psycholinguistic characteristics of the words used as deviant auditory stimuli in the cross-modal oddball task.

	Pons & Perpiñá (1996)		EsPal (Duchon et al., 2013)			
	Frequency	Familiarity	Log-Frequency	Familiarity	N syllables	Concreteness
Bombón	6.26 (2.75)	8.61 (2.01)	0.23	NA	2	NA
Helado	7.10 (2.66)	9.03 (1.81)	0.84	6.557	3	5.482
Natillas	6.07 (3.28)	8.33 (2.59)	0.10	NA	3	NA
Tarta	6.25 (2.84)	8.61 (2.24)	0.46	6.239	2	6.361
Pastel	6.70 (2.69)	8.63 (2.10)	0.74	6.437	2	5.859
M (SD)	6.48 (0.42)	8.64 (0.25)	0.47 (0.32)	6.41 (0.16)	2.40 (0.55)	5.9 (0.44)
Cajón	6.98 (2.52)	8.41 (1.97)	0.96	6.635	2	6.10
Cristal	6.71 (2.77)	9.26 (1.78)	1.43	6.638	2	5.88
Manta	8.23 (1.85)	9.26 (2.15)	0.83	6.701	2	6.09
Perchero	1.51 (0.93)	6.20 (1.73)	0.09	NA	3	NA
Persiana	7.44 (2.64)	9.05 (1.97)	0.28	5.788	3	6.28
M (SD)	6.17 (2.67)	8.44 (1.30)	0.72 (0.54)	6.44 (0.44)	2.40 (0.55)	6.09 (0.17)
t value	t(8) = 0.25	t(8) = 0.35	t(8) = 0.87	t(5) = 0.11	t(8) = 0	t(5) = 0.80
p value	.81	.74	.41	.92	1	.46

Participants were instructed to focus on the visual digit categorization task, to ignore the sounds, and to try to respond as quickly as possible while trying not to make errors. All data presented in this study are available from the Open Science Framework (https://osf.io/yvrdw/).

## Results and discussion

### Questionnaires

Participants in the food-deprived group did report a greater state of hunger (M = 7.458, SD = 1.581) than participants allocated to the satiated group (M = 1.5, SD = 1.041), t(46) = 15.099, p < .001. The time elapsed (measured in hours) since the last meal was significantly longer in the hungry group (M = 7.875, SD = 3.163) than in the satiated group (M = 0.972, SD = 1.303), t(46) = 9.678, p < .001.

The analysis of the measures related to food craving as a trait (FCQ-T) revealed no significant difference between hungry and satiated participants [t(46) = 1.34, p = .19], a result consistently observed across 9 of the 10 subscales (see Table 2).

**Table 2:** Mean scores on the FCQ-T and its subscales for hungry and satiated participants, and corresponding statistical comparisons.

	Hungry	Satiated	t(46)	p
Total score	116.29 (30.20)	106.42 (20.80)	1.32	0.19
Having intentions and plans to consume food	9.54 (3.39)	8.54 (2.62)	1.14	0.26
Anticipation of positive reinforcement that may result from eating	15.13 (4.25)	15.58 (3.31)	0.42	0.68
Anticipation of relief from negative states and feelings as a result of eating	8.42 (3.69)	8.00 (2.41)	0.46	0.65
Lack of control over eating	16.63 (5.37)	16.54 (4.84)	0.06	0.96
Thoughts or preoccupation with food	8.75 (3.74)	7.08 (2.92)	1.72	0.09
Having intentions and plans to consume food	7.29 (2.94)	6.33 (2.30)	1.26	0.21
Craving as a physiological state	15.42 (3.91)	14.67 (3.67)	0.69	0.50
Emotions that may be experienced before or during food cravings or eating	6.33 (2.14)	5.54 (2.17)	1.27	0.21
Cues that may trigger food cravings	14.33 (4.16)	12.13 (2.63)	2.20	0.03
Guilt from cravings and/or for giving in to them	8.75 (4.30)	6.75 (3.87)	1.69	0.10

Values within parentheses represent the standard deviation.

In contrast, as expected, the participants’ food craving state (FCQ-S) revealed greater craving in the hungry group compared to the satiated, both before the administration of the cross-modal oddball task [t(46) = 9.47, p < .001] and following it [t(46) = 9.53, p < .001]. These results were consistently observed across all of the FCQ’s subscales (see Table 3).

**Table 3:** Mean scores on the FCQ-S and its subscales for hungry and satiated participants, and corresponding statistical comparisons, prior to and following the administration of the cross-modal oddball task.

	Pre				Post			
	Hungry	Satiated	t(46)	p	Hungry	Satiated	t(46)	p
Total score	49.83 (12.61)	20.33 (8.60)	9.47	< .001	55.48 (12.31)	23.13 (11.18)	5.93	< .001
An intense desire to eat	10.71 (3.57)	4.13 (2.09)	10.71	< .001	11.26 (3.22)	4.78 (2.73)	7.49	< .001
Anticipation of positive reinforcement that may result from eating	9.50 (3.58)	4.58 (2.32)	5.65	< .001	10.78 (3.44)	5.04 (2.68)	6.46	< .001
Anticipation of relief from negative states and feelings as a result of eating	10.38 (3.25)	3.83 (1.97)	8.42	< .001	11.65 (2.73)	4.39 (2.60)	10.41	< .001
Lack of control over eating	7.08 (3.28)	4.17 (1.88)	3.78	< .001	8.7 (3.54)	4.57 (2.60)	4.60	< .001
Craving as a physiological state	12.17 (2.14)	3.63 (1.76)	15.09	< .001	13.09 (2.17)	4.35 (2.48)	13.02	< .001

Values within parentheses represent the standard deviation.

Both groups rated the food items as equally appealing [t(46) = 1.70, p = .10], a result that was consistent for all individual food items (see Table 4). Finally, hungry participants exhibited a significantly greater desire to eat the food items than satiated participants [t(46) = 4.55, p < .001], a difference observed for all individual food items (see Table 4). One-sample t-tests were conducted in order to compare the participants’ current desire to eat the food items to a neutral value of 25 (“5” rating x 5 food items). Hungry participants showed significant desire for the food [t(23) = 2.353, p = .028] while satiated participants showed significant aversion for it [t(23) = 3.961, p < .001].

**Table 4:** Mean food item appeal ratings and mean desire to eat the food items ratings for hungry and satiated participants, with statistical comparisons between these groups.

	Food item’s appeal				Current desire to eat the food items			
	Hungry	Satiated	t(46)	p	Hungry	Satiated	t(46)	p
All words	35.97 (5.97)	32.46 (8.16)	1.70	0.10	29.29 (8.96)	16.17 (10.92)	4.55	< .001
Bombón	7.86 (1.65)	6.83 (2.22)	1.84	0.07	6.13 (2.52)	3.67 (2.44)	3.62	< .005
Helado	7.96 (1.54)	7.13 (2.07)	1.55	0.13	6.25 (2.63)	3.71 (2.79)	3.25	< .005
Natillas	6.38 (2.14)	5.71 (2.12)	1.08	0.28	5.00 (2.84)	2.79 (2.60)	2.81	< .01
Pastel	6.46 (2.77)	6.13 (2.15)	0.47	0.63	5.79 (2.64)	3.00 (2.43)	3.82	< .001
Tarta	7.31 (1.77)	6.70 (2.03)	1.03	0.30	6.13 (2.46)	3.04 (2.65)	4.52	< .001

Values within parentheses represent the standard deviation.

In sum, data from the questionnaires showed that both groups exhibited similar levels of food craving as a trait and found the food items equally appealing in general. Hungry participants were in a greater state of food craving (both before and after the administration of the cross-modal oddball task) and experienced a greater desire to eat to food items than satiated participants. Finally, while hungry participants showed desire for the food, satiated participants exhibited aversion for it.

### Cross-modal oddball task

The mean proportion of correct responses and mean response times for correct responses were analyzed using 2 (group: hungry, satiated) x 2 (block: first, last) x 3 (sound: standard, food-related deviant, control-word deviant) ANOVAs. Response times shorter than 200ms longer than 800ms were treated as anticipations and abnormally slow responses respectively and excluded from the analysis (these represented 2.65% of the data).

**Fig 1:**
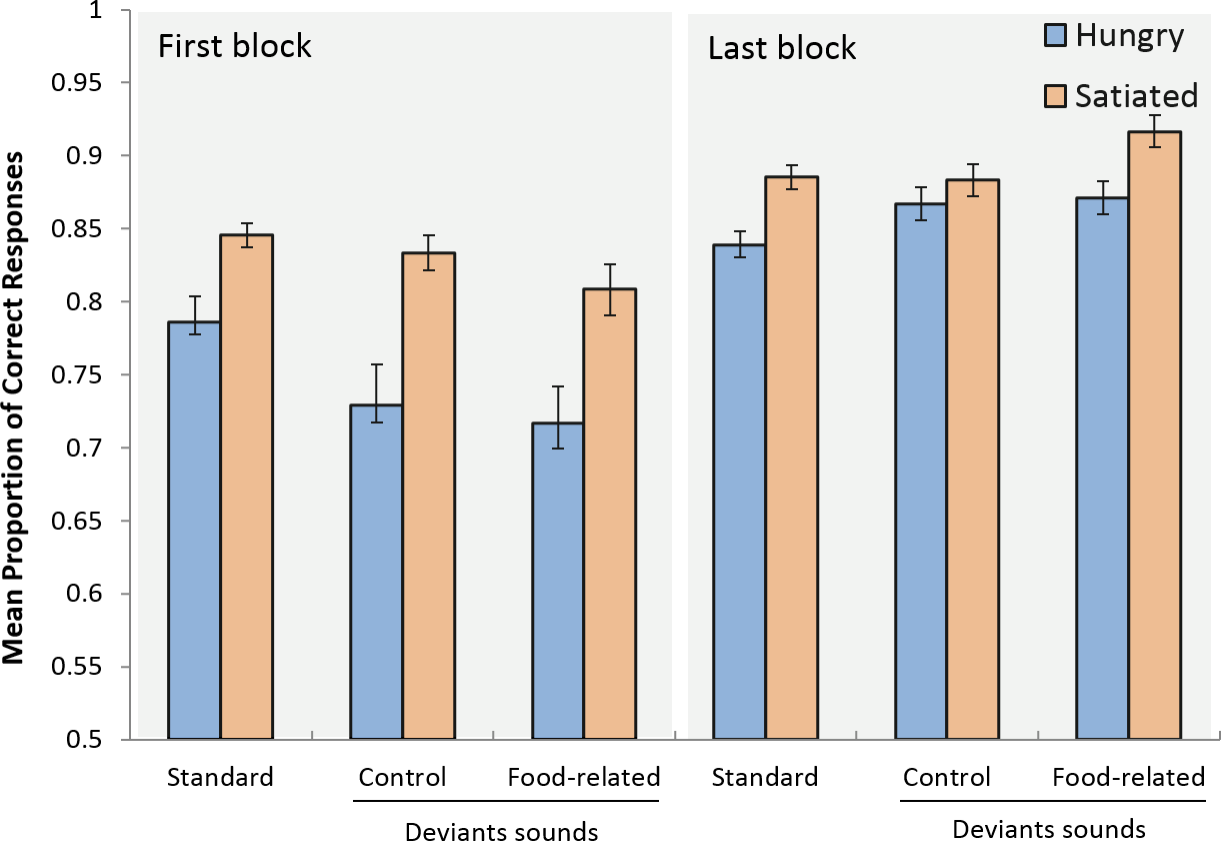
Mean proportion of correct responses as a function of the sound conditions, block and group. Error bars represent one standard error of the mean.

The analysis of the proportion of correct responses (see Fig 1) revealed a nearly significantly lower response accuracy in hungry compared to satiated participants [F(1,46) = 4.017, MSE = 0.066, p = .051, $\eta_{p}^{2}$ = 0.080], greater performance in the last block compared to the first [F(1,46) = 19.552, MSE = .030, p < .001, $\eta_{p}^{2}$ = 0.298]. No main effect of sound was observed [F(2,92) = 0.441, MSE = 0.008, p = .645, $\eta_{p}^{2}$ = 0.009]. The block x group and group x sound interactions were not significant [F(1,46) = 1.419, MSE = 0.030, p = .240, $\eta_{p}^{2}$ = 0.030; and F(2,92) = 0.173, MSE = 0.008, p = .814 $\eta_{p}^{2}$ = 0.004, respectively]. A significant block x sound interaction was found [F(2,92) = 4.278, MSE = 0.010, p = .017, $\eta_{p}^{2}$ = 0.085], reflecting the fact that performance was best in the standard condition and worst in the food-related condition in the first block and that this pattern was reversed in the last block. Finally, the 3-way interaction was not significant [F(2,92) = 0.827, MSE = 0.010, p = .441, $\eta_{p}^{2}$ = 0.018].

**Fig 2:**
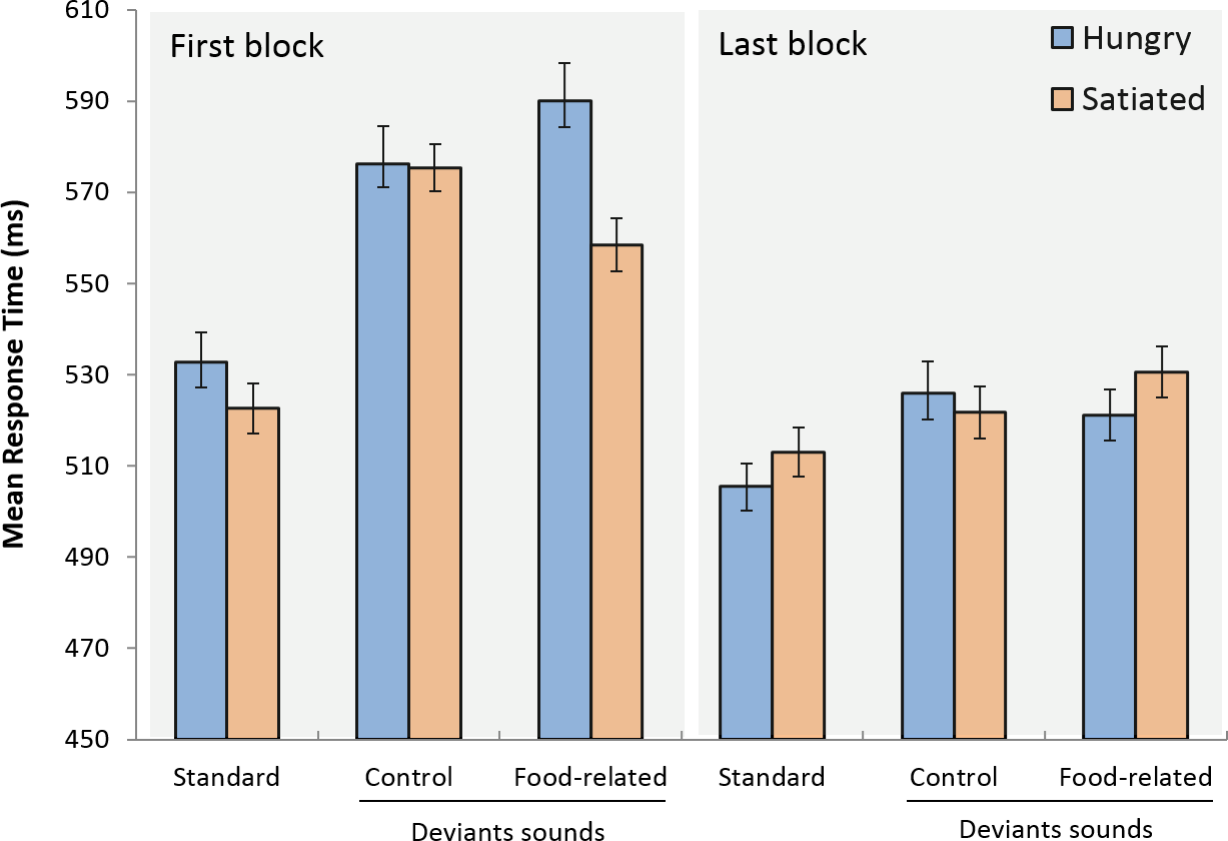
Mean response time for correct responses as a function of the sound conditions, block and group. Error bars represent one standard error of the mean.

The analysis of response times revealed no main effect of group [F(1,46) = 0.134, MSE = 14823, p = .716, $\eta_{p}^{2}$ = 0.003]. Response times were faster in the last block than in the first [F(1,46) = 34.382, MSE = 3225, p < .001, $\eta_{p}^{2}$ = 0.428], and varied with the sound condition [F(2,92) = 28.504, MSE = 1128, p < .001, $\eta_{p}^{2}$ = 0.383]. The group x block and group x sound interactions were not significant [F(1,46) = 1.762, MSE = 3225, p = .191, $\eta_{p}^{2}$ = 0.037; and F(2,92) = 0.551, MSE = 1128, p = .578, $\eta_{p}^{2}$ = 0.012, respectively]. A significant block x sound interaction was observed [F(2,92) = 8.341, MSE = 957, p < .001, $\eta_{p}^{2}$ = 0.153]. Importantly, the 3-way interaction was significant [F(2,92) = 3.319, MSE = 957, p = .041, $\eta_{p}^{2}$ = 0.067]. As visible from Fig 2, this 3-way interaction reflected differential distraction effects of food-related and control deviant words across the two groups in the first block relative to the last block. A contrast analysis comparing the two groups with respect to blocks and the type of deviant sound confirmed this observation [F(1,46) = 5.478, MSE = 1159.554, p = .024]. Further tests showed that food-related and control words had opposite effects for the two groups in the first block [F(1,46) = 4.419, MSE = 1266.969, p = .041] but not in the last block [F(1,46) = 1.276, MSE = 1125.547, p = .265]. In sum, in the first block, food-related deviant words increased distraction relative to control words in hungry participants, but had the opposite effect on satiated participants. These effects vanished in the last block.

As a complementary analysis, computed an index of the semantic effect (SE) by calculating the difference between, on the one hand, the difference between response times in the food-related (F) deviant and standard (S) conditions and, on the other hand, the difference between response times in the control (C) and standard (S) conditions:

SE=(F−S)−(C−S)

**Fig 3:**
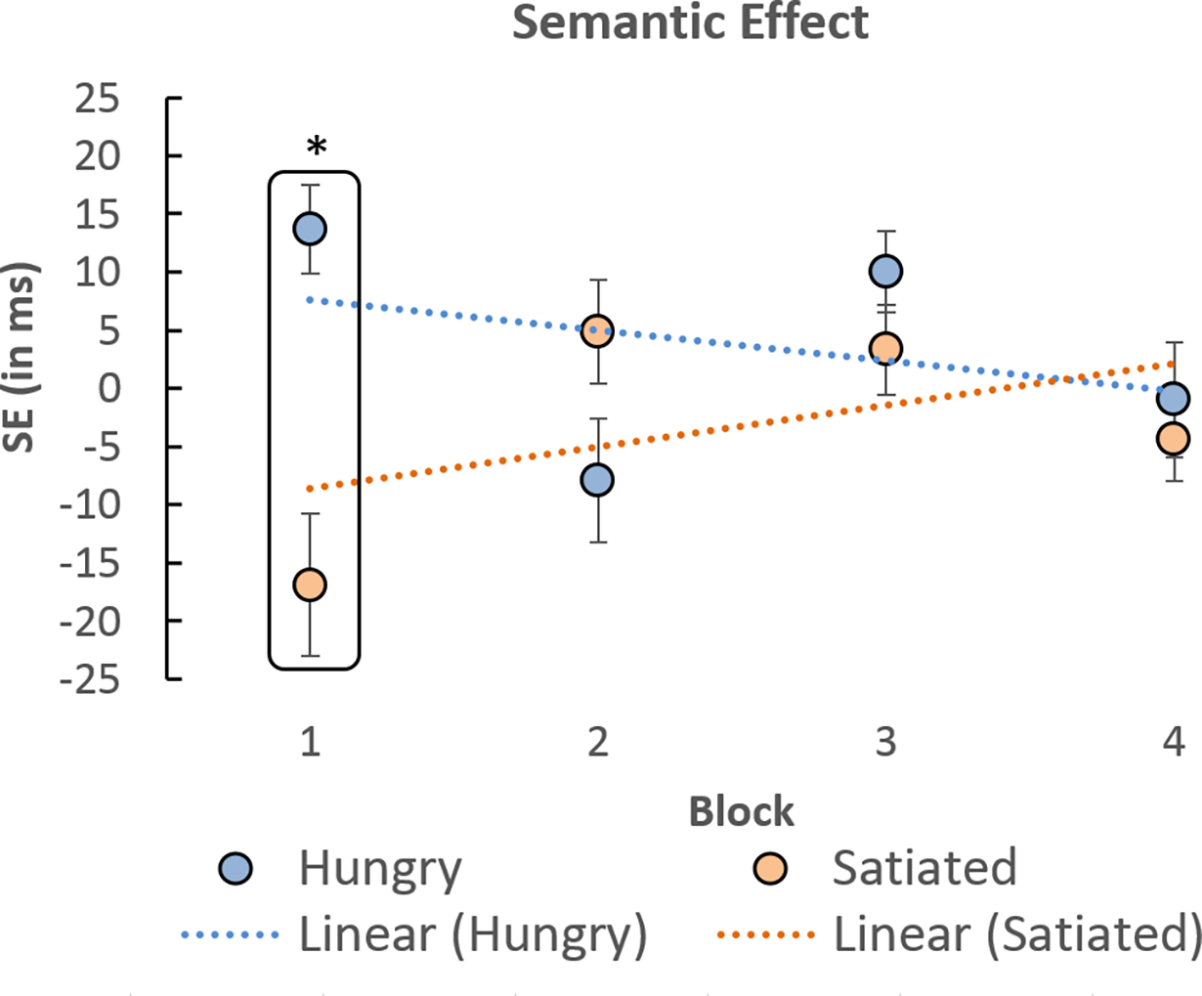
Effect induced by food-related deviant words relative to that induced by control deviant words, as a function of block and group. Dashed lines represent the linear trend exhibited by each groups across blocks. Error bars represent one standard error of the mean. * p < .05.

The SE index therefore reflects the amount of deviance distraction produced by food-related words relative to that induced by the control deviant words. As visible from Fig 3, comparisons between the hungry and satiated groups confirmed a significant difference in block 1 [t(46) = 2.102, p = .041] and the absence of difference between groups in blocks 2 to 4 [t(46) = 0.922, p = .361; t(46) = 0.647, p = .521; and t(46) = 0.283, p = .779, respectively].

Abundant evidence indicates that unexpected, task-irrelevant, sounds capture attention and yield a reduction in performance in a task at hand [2,3]. Past work suggests that these unexpected words undergo involuntary semantic analysis, which can in turn interfere with the processing of target stimuli in a primary task (e.g., [56]). Such evidence has however been restricted to a version of the cross-modal oddball task in which features of the unexpected sounds (words “left” or “right”) overlapped with relevant aspects of the primary task (categorizing left versus right visual arrows. Though evidence does indicate that electrophysiological markers of attention capture by unexpected sounds is modulated by the meaning or personal relevance of these sounds, their impact on behavior could so far not be clearly attributed to an automatic semantic evaluation unrelated to the primary task’s demands.

In this study we sought to address this issue by using unexpected auditory words that had no connection to the primary task’s feature but were, instead, of relevance to a biological need (food consumption). We did this by comparing the distractive effect of unexpected words related to food (relative to control words) in a cross-modal oddball task and found that food-related distractors yielded greater distraction in hungry participants than in satiated participants (in whom they were better ignored than control words). Such findings clearly demonstrate that the unexpected words were automatically processed even though they were entirely irrelevant for the task at hand.

Our method involved asking some participants to refrain from eating before taking part in the experiment, and to ask others to take part shortly after eating breakfast or lunch. All participants were tested at the same time of the day. While we had to rely on the participants’ good faith and compliance with these instructions, we took steps to maximize participants’ compliance by means of a mild deception, namely by informing them that we would take a saliva sample from them, which would then be submitted to a chemical analysis to estimate whether or not they had eaten recently. While this method is not bullet-proofed, the data from the FCQ-S and eating desire questionnaires, and most critically, the differential modulation of deviance distraction by food-related and control words across the two groups of participants, indicate that the manipulation was effective enough.

Relative to control words, food-related words yielded more distraction in hungry participants and less distraction in satiated participants, but this effect was only present in the first block. The disappearance of these effects cannot reasonably be attributed to a dissipation of hunger in the hungry group (for their score on the FCQ-S was numerically greater after the cross-modal oddball task than before) nor to the emerge of hunger in the satiated participants during the task (for their score on the FCQ-S was similar before and after the administration of the cross-modal oddball task, and their eating desire score following this task showed significant avoidance to the food items). The dissipation of these effects fits well with the notion of a saturation of the lexico-semantic activations. Such semantic satiation has been documented elsewhere [73–77,80] and described early on as a temporary lapse of meaning [78,79]. Such phenomenon is thought to reflect fatigue or adaptation in the neural activity underpinning meaning [73,74] or a reduced effectiveness of lexical representations to access meaning [80].

Prior work reported that the performance of participants performing a left/right categorization task is modulated by the presentation of the deviant words “left” and “right”, which demonstrates that the deviant words’ meaning is processed [56,58]. However, one limitation of these demonstrations was the overlap existing between the semantic features of the deviant words and the task requirements. Therefore, the cognitive system’s readiness to automatically process stimuli containing left/right information may be at least in part gated by the task-set voluntarily maintained by participants, in a way akin to the proposition that the maintenance of stimulus-response mappings and abstract task rules in the prefrontal cortex yield to the anticipation of sensory information or actions [84,85]. Our data show that the involuntary semantic processing of the deviant stimuli does occur in a situation in which distractors and task at hand do not share features and in which, therefore, such gating can be ruled out. Past electrophysiological studies did produce evidence of a semantic analysis of deviant stimuli using paradigms in which these stimuli bore no relationship to the subject’s primary task or in which participants were not performing any task at all. However, these studies did not report any corresponding behavioral effect. Examples include several studies suggesting that various aspects of novel or deviant stimuli, including their meaning, are picked up by the brain within 100-150ms of the sound’s onset in a situation where participants watch a silent movie [48,50]. Some have also argued that some degree of semantic processing occurs during sleep [86], although this contention is questioned [87]. Finally, variations in P3a have been observed as a function of the identifiability [54] or the aversive character of novel sounds [47] in tasks where these sounds’ meaning was unrelated to demands of the primary task (categorizing digits).

The results from our study extend past demonstrations of the involuntary appraisal of the deviant stimuli’s meaning [56–58] to a situation in which this meaning bares no connection to the task participants are performing. Instead, what we observed is that the participant’s biological needs, more specifically their state of hunger, modulated the extent to which food-related deviant words yielded distraction. As such, our results testify of the adaptive nature of the change detection mechanism. While both types of deviant stimuli (food-related and control words) distracted participants by virtue of their acoustic deviance [2,3], a state of hunger appears to modulate the attentional response to these stimuli. Further work will be necessary to ascertain the specific mechanisms underpinning this effect. One may hypothesize that biological necessities may exert some gating of sensory input in order to lower the detection threshold for items susceptible to address these necessities. A different, not mutually exclusive, possibility may be that such stimuli may be relatively difficult to disengage from, delaying the reorientation of attention to the primary task. It is also possible that the detection of stimuli addressing a biological need may potentiate an interruption of ongoing behavior in order to adopt a different action plan, which would then need to be disengaged from in order for participants to return to the task at hand. Finally, while our demonstration relied on a biological state (hunger), we expect that similar findings may be observed for other needs or indeed in relation to one’s personal goals or motivation.

## Conclusions

Unexpected deviations in an otherwise repetitive or structured sequence of task-irrelevant sounds capture attention and distract participants away from an ongoing task. Past work showed that the distractive effect of deviant words presented in the context of an ongoing visual categorization task is partially due to the incongruity between the involuntary semantic processing of the deviant sounds and the voluntary semantic processing of the visual target stimuli. However, past demonstrations relied on tasks in which the characteristics of the deviant sounds overlapped with aspects of the primary task demands (ignoring the deviant words “left” or “right” while categorizing left and right visual arrows). Here we sought to uncover evidence of the involuntary semantic processing of deviant words when they do not overlap with the demands of the primary task in any way but are, instead, of biological relevance to the participants. To achieve this, we compared hungry and satiated participants in a cross-modal oddball task in which we presented food-related and control deviant words. The results showed greater distraction by food-related deviant words compared to control deviant words in hungry participants, and the opposite patter of results in satiated participants. These effects were confined to the first block of the experiment, however, indicating that semantic saturation and desensitization to the deviant words’ meaning rapidly dampened the effect. Generally, the findings are indicative of the mediation of deviance distraction by the deviant words’ relation to the participants’ biological needs and of the involuntary semantic evaluation of auditory deviant words.
